# Synergistic antifungal evaluation of over-the-counter antifungal creams with turmeric essential oil or *Aloe vera* gel against pathogenic fungi

**DOI:** 10.1186/s12906-021-03205-5

**Published:** 2021-01-28

**Authors:** Clement Olusola Ogidi, Ayokunbi Elizabeth Ojo, Oluwatayo Benjamin Ajayi-Moses, Oluwatoyin Modupe Aladejana, Oluwakemi Abike Thonda, Bamidele Juliet Akinyele

**Affiliations:** 1 Biotechnology Unit, Department of Biological Sciences, Kings University, PMB 555, Odeomu, Nigeria; 2 Department of Microbiology, The Federal University of Technology, PMB 704, Akure, Nigeria; 3 Microbiology Unit, Department of Biological Sciences, Kings University, PMB 555, Odeomu, Nigeria

**Keywords:** Dermatophytes, -azole, Terbinafine, *Curcuma longa* rhizomes, Cosmeceutical, GC-MS

## Abstract

**Background:**

The frequent incidence of fungal infection and widespread of antibiotic resistance are emergent concerns in public health. Hence, there is a need to harness the potential of natural bioactive compounds from plant towards treatment of fungal infection. Combination effect of antibiotic creams with natural products from plants is prospective strategy to produce new antifungal agent. This study therefore, revealed antifungal effect of combined Antifungal Creams (AFCs) with Turmeric Essential Oil (TEO) or *Aloe vera* Gel (AVG).

**Methods:**

Phytochemicals and bioactive compounds in TEO and AVG were revealed using GC-MS. Bioactive compounds in plant extracts were compared to known compounds in database library of National Institute of Standards and Technology (U.S.). Antifungal activity and synergistic effect of AFCs with TEO or AVG were carried out using agar well diffusion method.

**Results:**

Phenol, flavonoids, saponins, alkaloids, steroids, terpenoids and cardiac glycosides were present in TEO and AVG. GCMS revealed thirty-six (36) and eighteen (18) bioactive compounds in TEO and AVG, respectively. AFCs displayed zones of inhibition with values ranged from 5.0 to 14.3 mm, TEO was 5.0 to 11.0 mm and AVG was 8.0 to 11.7 mm against tested fungi. Minimum Inhibitory Concentration (MIC) by AFCs, TEO and AVG ranged from 1.25 to 10.0 mg/ml. Combinatory effects of AFCs with TEO or AVG revealed synergistic and indifferent properties.

**Conclusion:**

Development of novel products using bioactive ingredients from plants with commercially available AFCs will serve as potential alternative therapy to cure dermatological infections with no side effects.

## Background

Mycotic diseases are causing significant morbidity and now seen as a serious concern to public health [1, 2]. The spread of fungal diseases is increasing by overuse of broad-spectrum antibiotics, which lessening non-pathogenic bacterial population that check the growth of fungi through competition [3]. Antifungal drugs play active roles in the treatment of some fungal infections but their misuse always made the fungal infection worsen. Superficial and subcutaneous fungal infections are very dangerous if not promptly and properly treated with appropriate drugs. The erroneous use of antifungal drugs has contributed to frequent resistance experience over the past decades [4]. In addition, most antifungal drugs currently available to treat fungal infections have serious drawbacks, which include low concentration of active ingredients, itching on the skin due to chemical composition, development of fungi resistance and toxic side effects [5]. Conventional formulation of creams, powder, gels to treat skin or deep seated fungal infections still have various side-effects like burning, redness and swelling on the application site [6]. Beside different side effects committed to commercially available antifungal drugs, these days, multiple drug resistance is fast rising worldwide and efficacy of single antibiotic against resistant microorganisms is abating. The continuous therapeutic failure as a result of multi-drug resistance by pathogenic fungi is urgently demanding for innovative complementary approach towards production of novel, alternative and effective antimicrobial drugs. Invention of antifungal drugs with different drug delivery systems like liposomes, niosomes, ethosomes, microemulsions, microsponge, nanoparticles are now embraced as treatment option for fungal infection in order to achieve clinical efficacy [6, 7]. The increasing therapeutic potentials of drug combination has been found more efficacious than single antifungal agent aimed at a single target [8].

To overcome the drawbacks of conventional therapy and to produce antifungal agents with dose efficient, a blueprint for development of effective antifungal drugs or creams from natural herbs is now an element of consensus among researchers, medical practitioners and pharmaceutical companies [9]. To enhance efficacies of antibiotics (creams or drugs) and to minimize their side effects, combinatory effect of commercial antifungal drugs with natural products will demonstrate a huge success in treating fungal infections. Antifungal drugs with live lactic acid bacteria (probiotics) were designed to protect, restore the natural balance in the vagina and help to fight yeast infection without side effects [10]. Globally, combination and synergistic interaction of antimicrobial agents with multiple herbs formulation of different natural bioactive compounds are the main therapy to cure some medical challenges [11]. Antifungal combination therapy is trending with veracity in the fields of infectious diseases and medical mycology [12]. The combination and synergistic effects of commercial antifungal drugs with natural bioactive compounds from plants will be a complementary and alternative approach to combat reoccurrence incidences of fungal infection.

Medicinal plants are imperious sources of treasurable secondary metabolites and thus, contribute to availability of natural drugs in global markets [13]. Turmeric; *Curcuma longa* belong to ginger family; Zingiberaceae. Turmeric powder from dried ground rhizomes of *C. longa* possess culinary uses, act as natural product with analgesic, antibacterial, antifungal, anti-inflammatory, antioxidant, and digestive properties. Hence, biological activities of turmeric, its health promoting effects and disease prevention have attracted several applications in pharmaceutical, food and biotechnological industries [14]. *Aloe vera,* is another medicinal plant with immense bioactive compounds allied with some pharmacological properties like wound healing, antifungal activity, hypoglycemic or antidiabetic effects, anti-inflammatory, anticancer, immunomodulatory and gastroprotective [15]. The therapeutic potentials of *A. vera* in phytomedicine indicate its several uses in pharmaceutical and cosmetic industries [16]. Natural products in plants act as reservoirs of novel bioactive compounds and as excellent source of drug discovery with divers’ biopharmaceutical applications [17], hence, biologically active compounds in medicinal plants can be exploited and incorporated in AFCs, which will be a newsworthy option towards new prototype antifungal agents. This study therefore, revealed synergistic antifungal potential of over-the-counter AFCs with TEO or AVG against clinically important pathogenic fungi.

## Methods

### Sample collection

Turmeric was collected from a farmland in Isarun Village, Ifedore Local Government Area, Ondo State. The rhizomes were washed with distilled water before they were cut into smaller pieces. *Aloe vera* was obtained from a farmland in Aule, Akure South Local Government Area, Ondo state. *C. longa* rhizomes and *Aloe vera* were authenticated at the Department of Crop, Soil and Pest Management, The Federal University of Technology, Akure and was deposited in the herbarium of the same Department.

### Source of antifungal creams (AFCs)

The commercially available AFCs namely; clotrimazole (1%), fluconazole (0.5%), ketoconazole (2%) and terbinafine (1%) were purchased. These AFCs were certified by National Agency for Food and Drug Administration and Control (NAFDAC), a federal agency under the Federal Ministry of Health; responsible for regulation and control of importation, exportation, advertisement, distribution, sale and use of food, drugs, cosmetics, medical devices, chemicals and packaged water in Nigeria.

### Collection of tested fungi

The tested fungal isolates namely: *Candida tropicalis* (ATCC 66029) was obtained from Nigeria Institute of Medical Research, Lagos. *Candida albicans, Penicillium notatum, Aspergillus fumigatus*, *A. niger, A. flavus, Trichophyton rubrum, Trichophyton violceum* and *Trichophyton mentagrophytes* were collected from Department of Medical Microbiology Laboratory, Federal Medical Centre, Ido-Ekiti, Nigeria.

### Extraction of TEO and AVG

Essential oil was extracted from the turmeric rhizomes by the process of steam distillation using Clevenger apparatus [18]. Fresh rhizome (100 g) of turmeric was boiled with 500 ml of distilled water in a Clevenger apparatus until oil distillation ceased after 5 h. The volume of essential oil was determined from a calibrated trap. The essential oil in the distillate were dried over anhydrous Na_2_SO_4_ and kept in the freezer. *A. vera* leaf was cleaned with ethanol, dissected and the gel; jelly-like substance found in the inner part of the *A. vera* leaf was aseptically collected into sterile tubes.

### Determination of phytochemicals and bioactive compounds in TEO and AVG

Qualitative and quantitative phytochemicals in TEO and AVG were determined using the standard methods. Briefly, total phenolic and flavonoid contents of extracts was determined according to the method of Sofowora [19]. The methods stated by Harborne [20] and Trease and Evan [21] were used to determined alkaloids, saponins, tannins, steroid, tepernoids and cardiac glycoside. The method described by Soladoye [22] was used to determine the anthraquinone content.

The bioactive compounds in the TEO and AVG were identified with the aid of gas chromatography– mass spectrometry (QP2010 plus Shimadzu, Japan), which was equipped with a split injector and an ion – trap mass spectrometer detector together with a fused – silica capillary column having a thickness of 1.00 μm, dimensions of 20 m × 0.22 mm and temperature limits of 60 °C to 325 °C. The column temperature was programmed between 60 °C and 250 °C at a rate of 0.5 m/s with pressure of 100.2 Kpa. The temperature of the injector and detector were at 250 °C and 200 °C respectively. Helium gas was used as a carrier gas at flow rate of 0.46 m/s. The MS analysis was done based on comparative retention times, mass and peaks of the chemical compounds using the computer-aided matching of unknown mass spectra of compounds with the known compounds stored in the software database library from the National Institute of Standards and Technology (NIST), Washington, USA, having more than 62,000 patterns as the reference database. The name, molecular weight and the structure of the components of the tested materials were ascertained with database library from the NIST, Washington, USA.

### Antifungal activities of AFCs, TEO and AVG

The antifungal assay was carried out using the agar well diffusion method described by CLSI [23, 24]. Suspensions of fungi (1.0 × 10^5^ sfu/ml) was adjusted with the aid of spectrophotometer (UNICO S-1100 RS) to 0.5 McFarland standard. Dimethyl sulfoxide (DMSO 2% v/v) was used to reconstitute since most of AFCs and plant extracts (TEO and AVG) were not soluble in sterile distilled water. The concentration of AFCs, TEO and AVG were reconstituted to 10.0 mg/ml. Plant extract was sterilized using a Millipore membrane filter (0.22 μm). The sterility of TEO and AVG were confirmed after Millipore filtration, by introducing 0.1 ml of supposed sterile extract into sterilized nutrient agar and potato dextrose agar. Each labelled plate was seeded with tested fungi by means of sterile swab stick rolled on potato dextrose agar. Sterile cork borer was used to make well (6 mm) in the Petri dishes. Aliquots of TEO, AVG and AFCs (50 μl) were dropped in each well. DMSO solution was used as the negative control. The plates were incubated at 26 °C for 48 h. The zones of inhibition around well were measured in millimeter (mm). For synergism activity, concentration of each AFCs, TEO and AVG was adjusted to 3.0 mg/ml.

### Determination of minimum inhibitory and fractional inhibitory concentration index (FICi)

The minimum inhibitory concentration was determined by using method described by CLSI [23, 24].

The varying concentrations of 1.25, 2.5, 5.0, and 10.0 mg/ml for AFCs, TEO and AVG were prepared and incorporated into a set of sterile tubes. Each test tube was inoculated with 0.1 ml of standardized fungal inoculum and incubated at 26 °C for 48 h. The MIC were recorded as the lowest concentration to prevent growth of macroscopically visible colonies on plates, while there was visible growth on plates without AFCs, TEO and AVG. To determine MIC of combined AFC with TEO or AVG, varying concentrations of 1.0–3.0 mg/ml was used. The synergism, indifference, and antagonism of combined AFC with TEO or AVG were screened on the studied pathogenic fungi. MICs were transformed into Fractional Inhibitory Concentration (FIC) to determine the interaction of two samples in the following manner:

FIC of AFC = MIC of AFC in presence of TEO/MIC of AFC

FIC of TEO = MIC of TEO in presence of AFC /MIC of TEO

or

FIC of AFC = MIC of AFC in presence of AVG /MIC of AFC

FIC of AVG = MIC of AVG in presence of AFC /MIC of AVG

Fractional Inhibitory Concentration index (FICi) for each sample was calculated from FIC values as follows:

FICi = FIC of AFC + FIC of TEO

or

FICi = FIC of AFC + FIC of AVG.

The FICi was interpreted as: synergistic when FICi ≤0.5; indifferent when FICi was 0.5–4.0 and antagonistic when FICi ≥4.0 [25].

### Statistical analysis

Experimental studies were carried out in replicates (*n* = 3). Data obtained were subjected to one-way analysis of variance (ANOVA) using Statistical Package for Social Sciences (SPSS) version 20, USA. Results obtained were reported as mean ± standard deviation (SD). Values were compared by Duncan’s new multiple range test (MRT) and differences were considered significant when *P* < 0.05.

## Results

### Phytochemical and bioactive compounds in TEO and AVG as revealed by GC-MS

**Fig. 1:**
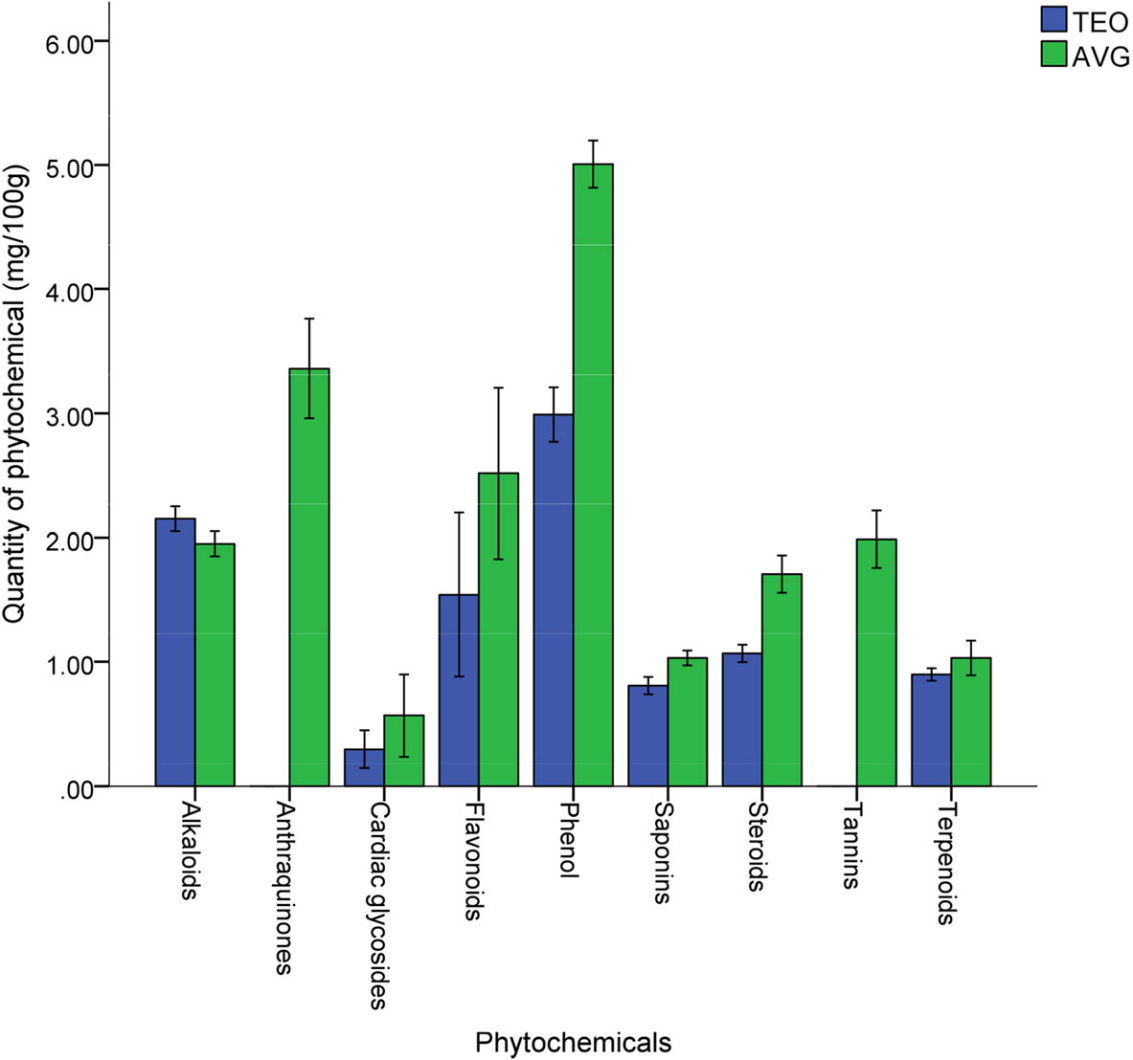
Quantitative constituents (mg/100 g) of phytochemicals in TEO and AVG. Error bar is SD

**Fig. 2:**
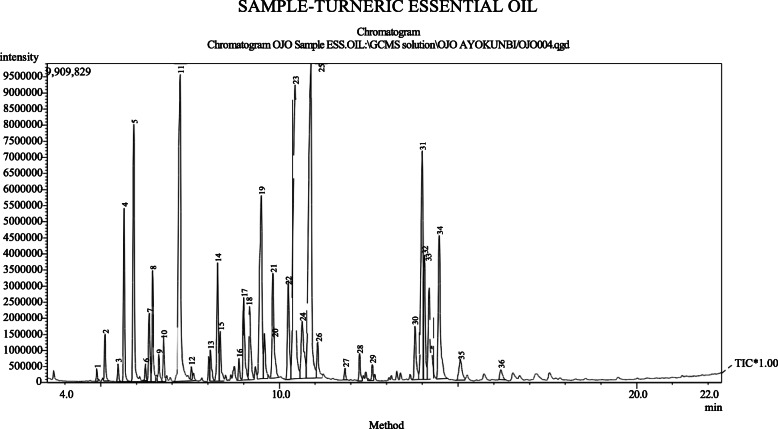
Chromatogram of TEO with peaks for various bioactive constituents

**Fig. 3:**
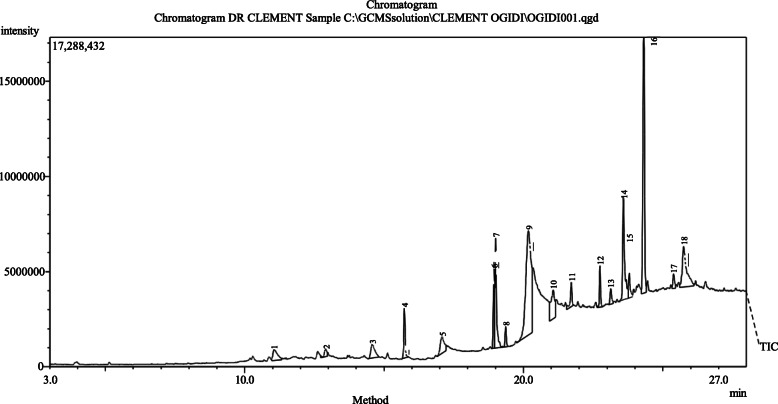
Chromatogram of AVG with peaks for various bioactive constituents

The quantity of phytochemicals of TEO and AVG was revealed in Fig. 1. Phytochemical such as phenol, flavonoids, saponins, alkaloids, steroids, terpenoids and cardiac glycosides were present in TEO and AVG. while anthraquinones and tannins was present only in AVG. Phenol was present with values of 2.9 mg/ 100 g and 5.1 mg/ 100 g in TEO and AVG, respectively. Alkaloid in TEO and AVG were not significantly different (*p* = 0.05) with value of 2.3 mg/100 g and 2.1 mg/100 g. Anthraquinones and tannins in AVG was 3.5 mg/100 g and 2.3 mg/100 g, respectively. Cardiac glycoside has the least values of 0.30 mg/100 g in TEO and 0.48 mg/100 g in AVG. Figures 2 and 3 show chromatogram of TEO and AVG with peaks for various bioactive constituents. The peaks were shown for 36 and 18 bioactive compounds in TEO and AVG, respectively. Tables 1 and 2 show the presence of bioactive compounds in TEO and AVG, respectively identified with GC MS. Z-citral was the major compound in the TEO (14.02%), while Z-9-Tetradecenol (24.99%) was the most abundant compound in AVG. Bioactive compounds such as α -pinene, camphene, linalool, borneol, p-menth-1-en-8-ol, zingiberene, farnesene, farnesol and others were found in TEO (Table 1). In AVG, cis oleic acid, dioctyl adipraate, glycerin 1,3-distearate, arachidic acid methyl ester, dipentene diepox, z-9-tetradecenol and others (Table 2).

**Table 1:** Main component and the relative contents of TEO as revealed by GCMS

Peaks	Retention time	Area %	Bioactive compounds	Molecular formula	Molecular weight
1	4.886	0.19	n-pentyl methyl ketone	C_7_H_14_O	114
2	5.115	0.85	Amyl methyl carbinol	C_7_H_16_O	116
3	5.483	0.30	Tricyclene	C_10_H_16_	136
4	5.652	3.01	α -pinene	C_10_H_16_	136
5	5.924	6.25	Camphene	C_10_H_16_	136
6	6.251	0.25	Sabinene	C_10_H_16_	136
7	6.355	1.55	6-Methyl-5-hepten-2-one	C_8_H_14_O	126
8	6.452	2.01	β -Myrcene	C_10_H_16_	136
9	6.625	0.55	Caprylaldehyde	C_8_H_16_O	128
10	6.756	0.82	α-Phellandrene	C_10_H_16_	136
11	7.222	11.41	cis-.beta.-Terpineol	C_10_H_18_O	154
12	7.535	0.23	(E)-2-Octenal	C_8_H_14_O	126
13	8.070	0.76	2-Carene	C_10_H_16_	136
14	8.272	2.57	Linalol	C_10_H_18_O	154
15	8.341	1.21	d-Verbenol	C_10_H_16_O	152
16	8.871	0.41	2-Methyl- 6-methylene 2-octene	C_10_H_18_	138
17	9.001	2.18	Citronellal	C_10_H_18_O	154
18	9.167	1.90	Artemiseole	C_10_H_16_O	152
19	9.494	6.59	Borneol	C_10_H_18_O	154
20	9.584	1.09	1-Terpinen-4-ol	C_10_H_18_O	154
21	9.818	3.10	p-menth-1-en-8-ol	C_10_H_18_O	154
22	10.248	2.65	β -Citronellol	C_10_H_20_O	156
23	10.440	11.94	cis,trans-Citral	C_10_H_16_O	152
24	10.635	2.48	trans-Geraniol	C_10_H_18_O	154
25	10.881	14.02	Z-Citral	C_10_H_16_O	152
26	11.072	1.06	Methyl nonyl ketone	C_10_H_18_	138
27	11.836	0.21	(6E)-2,6-Dimethyl-2,6-octadiene	C_10_H_18_	138
28	12.247	0.57	Geraniol acetate	C_12_H_20_O_2_	172
29	12.603	0.34	2,4-Diisopropenyl-1-methyl-1-vinylcyclohexane	C_15_H_24_	204
30	13.794	1.54	α-Curcumene	C_15_H_22_	202
31	13.998	7.27	Zingiberene	C_15_H_24_	204
32	14.056	2.68	Farnesene	C_15_H_24_	204
33	14.189	2.26	(Z)-.beta.-Farnesene	C_15_H_24_	204
34	14.472	4.47	β-Sesquiphellandrene	C_15_H_24_	204
35	15.064	0.92	D-nerolidol	C_15_H_26_O	222
36	16.196	0.37	(2E,6E)-Farnesol	C_15_H_26_O	222

**Table 2:** Main component and the relative contents of AVG as revealed by GCMS

Peaks	Retention time	Area %	Bioactive compounds	Molecular formula	Molecular weight (g/mol)
1	11.049	2.18	3-Hydroxybenzhydrazide	C_7_H_8_N_2_O_2_	167
2	12.905	0.88	4-Decyl methylphosphonofluoridate	C_11_H_24_FO_2_P	182
3	14.574	2.49	Dipentene diepox	C_10_H_16_O_2_	136
4	15.727	3.23	Arachidic acid methyl ester	C_21_H_42_O_2_	296
5	17.08	3.05	1-Pentadecanecarboxylic acid	C_16_H_32_O_2_	314
6	18.941	4.78	Methyl (9E,12E)-9,12-octadecadienoate	C_19_H_34_O_2_	294
7	19.00	7.89	Methyl (10E)-10-octadecenoate	C_19_H_36_O_2_	296
8	19.36	1.19	Methyl arachisate (Kemester 2050)	C_21_H_42_O_2_	326
9	20.17	24.99	Z-9-Tetradecanol	C_14_H_26_O	214
10	21.07	4.99	trans-13-Docosenoic acid	C_22_H_42_O_2_	338
11	21.71	1.86	Glycerin 1,3-distearate	C_39_H_76_O_5_	625
12	22.74	2.10	Dioctyl adipate	C_22_H_42_O_4_	370
13	23.12	1.15	Lineoleoyl chloride	C_18_H_31_ClO	300
14	23.58	9.12	cis-Oleic acid	C_18_H_34_O_2_	282
15	23.79	1.98	Eicosanoic acid	C_20_H_40_O_2_	312
16	24.31	18.99	n-Octyl phthalate	C_24_H_38_O_4_	390
17	25.38	1.12	13-Tetradecenal	C_14_H_26_O	210
18	25.74	8.00	Z-9-Tetradecenol	C_14_H_26_O	212

**Table 3:** Zones of inhibition (mm) by AFCs, TEO and AVG against pathogenic fungi at 10.0 mg/ml of each tested agent

Isolates	Clotrimazole	Fluconazole	Ketoconazole	Terbinafine	TEO	AVG
*Candida tropicalis* (ATCC 66029)	10.6 ± 0.2 ^a^	8.0 ± 0.0 ^b^	11.0 ± 0.0^a^	8.0 ± 0.1 ^b^	10.0 ± 0.1 ^a^	8.3 ± 0.0 ^b^
*Candida albicans*	5.3 ± 0.0 ^c^	7.0 ± 0.6 ^b^	9.0 ± 0.0 ^a^	9.3 ± 0.0 ^a^	5.0 ± 0.0 ^c^	9.7 ± 0.1 ^a^
*Penicillium notatum*	11.6 ± 0.1 ^a^	11.3 ± 0.6 ^a^	7.0 ± 0.3 ^b^	10.7 ± 0.3 ^a^	10.0 ± 0.3 ^a^	10.0 ± 0.0 ^a^
*Aspergillus fumigatus*	8.0 ± 0.0 ^b^	6.0 ± 0.0 ^c^	5.0 ± 0.0 ^c^	11.0 ± 0.0 ^a^	9.2 ± 0.0 ^ab^	10.0 ± 0.0 ^a^
*Aspergillus niger*	11.0 ± 0.2 ^a^	8.0 ± 0.0 ^c^	7.3 ± 0.1 ^c^	10.0 ± 0.0 ^ab^	8.3 ± 0.0 ^c^	12.7 ± 0.2 ^a^
*Aspergillus flavus*	10.6 ± 0.2 ^b^	9.0 ± 0.5 ^bc^	6.0 ± 0.0 ^d^	14.0 ± 0.0 ^a^	11.0 ± 0.0 ^b^	8.0 ± 0.0 ^c^
*Trichophyton rubrum*	5.0 ± 0.0 ^c^	5.3 ± 0.0 ^c^	10.3 ± 0.1^b^	14.3 ± 0.2 ^a^	10.3 ± 0.2 ^b^	11.7 ± 0.3 ^b^
*Trichophyton violceum*	8.0 ± 0.1 ^b^	6.0 ± 0.0 ^c^	11.3 ± 0.0 ^a^	10.6 ± 0.3 ^a^	10.6 ± 0.3 ^a^	8.7 ± 0.1 ^b^
*Trichophyton mentagrophytes*	8.3 ± 0.0 ^b^	5.0 ± 0.0 ^d^	8.0 ± 0.0 ^b^	12.7 ± 0.3 ^a^	7.9 ± 0.0 ^bc^	9.0 ± 0.5 ^b^

Value are mean ± SD of replicates (*n* = 3). Values with the same superscript alphabet along row are not significantly different from each other when *P* < 0.05

**Table 4:** Zones of inhibition (mm) displayed by combined AFCs with TEO or AVG against pathogenic fungi at 3.0 mg/ml of each tested agent

Isolates	Clotrimazole + TEO	Fluconazole + TEO	Ketoconazole + TEO	Terbinafine + TEO	Clotrimazole + AVG	Fluconazole + AVG	Ketoconazole + AVG	Terbinafine + AVG
*C. tropicalis* (ATCC 66029)	9.6 ± 0.2 ^bc^	8.0 ± 0.0 ^c^	10.0 ± 0.0 ^a^	12.0 ± 0.3 ^a^	7.7 ± 0.0 ^cd^	10.3 ± 1.5 ^a^	7.3 ± 0.1 ^cd^	11.3 ± 1.2 ^a^
*C. albicans*	10.6 ± 0.2 ^b^	11.0 ± 0.5 ^b^	9.0 ± 0.0 ^c^	8.6 ± 0.8 ^c^	9.0 ± 1.0 ^c^	8.0 ± 2.0 ^c^	7.0 ± 0.0 ^d^	12.7 ± 1.0 ^a^
*Penicillium notatum*	10.7 ± 0.6 ^b^	11.3 ± 0.6 ^b^	7.7 ± 0.3 ^d^	9.7 ± 0.3 ^c^	9.0 ± 1.0 ^c^	6.0 ± 1.0 ^e^	8.0 ± 1.0 ^d^	13.6 ± 1.3 ^a^
*A. fumigatus*	9.0 ± 0.6 ^b^	8.0 ± 0.0 ^b^	8.0 ± 0.0 ^b^	8.0 ± 0.0 ^b^	11.0 ± 2.0 ^a^	11.0 ± 1.0 ^a^	10.6 ± 1.7 ^a^	10.7 ± 0.5 ^a^
*A. niger*	10.0 ± 0.6 ^a^	8.0 ± 0.0 ^b^	7.0 ± 0.0 ^b^	7.0 ± 0.0 ^b^	7.0 ± 1.0 ^b^	7.7 ± 0.5 ^b^	8.6 ± 0.3 ^b^	11.3 ± 0.3 ^a^
*A. flavus*	9.6 ± 0.2 ^b^	7.0 ± 0.0 ^c^	9.0 ± 0.5 ^b^	11.0 ± 0.5 ^a^	10.0 ± 1.0 ^a^	9.0 ± 1.0 ^b^	7.3 ± 0.3 ^c^	10.7 ± 0.5 ^a^
*T. rubrum*	6.0 ± 0.5 ^d^	10.3 ± 0.3 ^b^	11.0 ± 0.5 ^b^	9.6 ± 0.3 ^bc^	8.0 ± 0.3 ^c^	10.7 ± 1.3 ^b^	8.0 ± 0.0 ^c^	13.7 ± 0.3 ^a^
*T. violceum*	10.0 ± 0.6 ^a^	9.3 ± 0.0 ^ab^	9.0 ± 0.5 ^ab^	10.3 ± 1.0 ^a^	10.0 ± 0.0 ^a^	8.0 ± 1.0 ^b^	11.3 ± 0.5 ^a^	10.0 ± 0.0 ^a^
*T. mentagrophytes*	8.0 ± 0.5 ^cd^	9.0 ± 0.5 ^c^	8.3 ± 0.3 ^c^	7.6 ± 0.3 ^d^	11.7 ± 1.1 ^b^	10.0 ± 2.0 ^b^	12.7 ± 0.0 ^a^	13.7 ± 0.8 ^a^

Value are mean ± SD of replicates (*n* = 3). Values with the same superscript alphabet along row are not significantly different from each other when *P* < 0.05

### Inhibitory potentials and synergistic antifungal efficacy of AFCs with TEO or AVG against pathogenic fungi

The zones of inhibition (mm) reflecting the antifungal efficacy of AFCs, TEO and AVG were reported in Table 3. AFCs namely; clotrimazole, fluconazole, ketoconazole and terbinafine displayed zones of inhibition against tested fungi with values ranged from 5.0 to 11.6 mm, 5.0 to 11.3 mm, 5.0 to 11.3 mm and 8.0 to 14.3 mm, respectively. TEO have inhibitory zones of 5.0 to 11.0 mm, while AVG have 8.0 to 11.7 mm against tested fungi. Varying zones of inhibition indicated antifungal activity of combined AFCs with TEO or AVG as shown in Table 4. Combinatory effect of AFCs with TEO or AVG showed better inhibitory zones against fungi. Ketoconazole + TEO, terbinafine + TEO, fluconazole + AVG and terbinafine + AVG have similar (*p* < 0.05) inhibitory effects against *C. tropicalis* (ATCC 66029). Terbinafine + AVG displayed the highest (*p* < 0.05) zones of inhibition of 12.7 mm and 13.6 mm against *C. albicans* and *Penicillium notatum*, respectively. Zones of inhibition displayed by each AFC combined with AVG against *A. fumigatus* were not significantly different when *p* < 0.05. Inhibitory action of each AFC combined with TEO against *A. fumigatus* were also similar. Ketoconazole + AVG and terbinafine + AVG respectively have similar inhibitory zones of 12.7 mm and 13.7 mm against *T. mentagrophytes*.

Table 5 shows minimum inhibitory concentration of AFCs, TEO and AVG against fungi. AVG displayed lower range of MIC values (1.25 to 5.0 mg/ml), while other were within 1.25 to 10 mg/ml. The MIC obtained for combined AFCs with TEO or AVG was shown in Table 6. Combination of clotrimazole + AVG displayed lower MIC value of 1.0 to 2.0 mg/ml against tested fungi. The MICs of fluconazole + AVG, ketoconazole + AVG, and terbinafine + AVG were within 1.0 to 2.5 mg/ml against tested fungi. Table 7 shows FIC, FIC indices (FICi) as well as their interpretation. Clotrimazole + TEO against *C. albicans,* ketoconazole + TEO against *A. niger,* terbinafine + TEO against *C. albicans*, clotrimazole + AVG against *C. albicans,* fluconazole + TEO against *A. flavus* and terbinafine + AVG against *C. tropicalis* (ATCC 66029) displayed synergistic properties. Other combinatory effects of AFC with TEO or AVG were indifferent without antagonism.

**Table 5:** Minimum inhibitory concentration (mg/ml) of AFCs, TEO and AVG against pathogenic fungi

Isolates	Clotrimazole	Fluconazole	Ketoconazole	Terbinafine	TEO	AVG
*C. tropicalis* (ATCC 66029)	2.50	2.50	5.00	10.00	2.50	2.50
*C. albicans*	10.00	5.00	5.00	10.00	10.00	5.00
*P. notatum*	2.50	2.50	5.00	2.50	5.00	2.50
*A. fumigatus*	5.00	5.00	10.00	2.50	5.00	5.00
*A. niger*	5.00	5.00	10.00	2.50	10.00	2.50
*A. flavus*	2.50	5.00	10.00	1.25	5.00	5.00
*T. rubrum*	10.00	10.00	5.00	2.50	5.00	2.50
*T. violceum*	10.00	10.00	5.00	1.25	5.00	1.25
*T. mentagrophytes*	5.00	10.00	5.00	5.00	5.00	2.50

**Table 6:** Minimum inhibitory concentration (mg/ml) of combined AFCs with TEO or AVG against pathogenic fungi

Isolates	Clotrimazole + TEO	Fluconazole + TEO	Ketoconazole + TEO	Terbinafine + TEO	Clotrimazole + AVG	Fluconazole + AVG	Ketoconazole + AVG	Terbinafine + AVG
*C. tropicalis* (ATCC 66029)	1.50	1.00	2.00	3.00	1.00	1.50	1.00	1.00
*C. albicans*	2.50	3.00	2.00	2.00	1.00	2.50	2.00	2.00
*P. notatum*	2.00	2.00	1.50	1.00	1.00	1.00	1.50	1.50
*A. fumigatus*	2.50	2.50	3.00	1.00	1.50	1.50	2.50	1.00
*A. niger*	2.00	3.00	2.50	1.50	1.00	2.50	1.50	1.50
*A. flavus*	2.00	2.50	2.50	1.50	1.50	1.00	2.00	1.50
*T. rubrum*	3.00	3.00	1.50	2.50	1.50	1.50	2.50	2.50
*T. violceum*	2.50	2.00	2.50	1.00	1.00	2.00	1.50	2.00
*T. mentagrophytes*	1.50	2.50	3.00	2.00	2.00	2.00	2.00	1.50

**Table 7:** Fractional inhibitory concentration (FIC) and FIC indices (FICi)

FIC ^a^FICi/interpretation	*C. tropicalis* (ATCC 66029)	*C. albicans*	*P. notatum*	*A. fumigatus*	*A. niger*	*A. flavus*	*T. rubrum*	*T. violceum*	*T. mentagrophytes*
Clotrimazole	0.60	0.25	0.80	0.50	0.40	0.80	0.30	0.25	0.30
TEO	0.60	0.25	0.40	0.50	0.20	0.40	0.60	0.50	0.30
**FICi**	**1.20/ind**	**0.50/syn**	**1.20/ind**	**1.00/ind**	**0.60/ind**	**1.20/ind**	**0.90/ind**	**0.75/ind**	**0.60/ind**
Fluconazole	0.40	0.60	0.80	0.50	0.60	0.50	0.30	0.20	0.25
TEO	0.40	0.30	0.40	0.50	0.30	0.50	0.60	0.40	0.50
**FICi**	**0.80/ind**	**0.90/ind**	**1.20/ind**	**1.00/ind**	**0.90/ind**	**1.00/ind**	**0.90/ind**	**0.60/ind**	**0.75/ind**
Ketoconazole	0.40	0.40	0.30	0.30	0.25	0.25	0.30	0.50	0.60
TEO	0.80	0.20	0.30	0.60	0.25	0.50	0.30	0.50	0.60
**FICi**	**1.20/ind**	**0.60/ind**	**0.60/ind**	**0.90/ind**	**0.50/syn**	**0.75/ind**	**0.60/ind**	**1.00/ind**	**1.20/ind**
Terbinafine	0.30	0.20	0.40	0.40	0.60	1.20	1.00	0.80	0.40
TEO	1.20	0.20	0.20	0.20	0.15	0.30	0.50	0.20	0.40
**FICi**	**1.50/ind**	**0.40/syn**	**0.60/ind**	**0.60/ind**	**0.75/ind**	**1.50/ind**	**1.50/ind**	**1.00/ind**	**0.80/ind**
Clotrimazole	0.40	0.10	0.40	0.30	0.20	0.60	0.15	0.10	0.40
AVG	0.40	0.20	0.40	0.30	0.40	0.30	0.60	0.80	0.80
**FICi**	**0.80/ind**	**0.30/syn**	**0.80/ind**	**0.60/ind**	**0.60/ind**	**0.90/ind**	**0.75/ind**	**0.90/ind**	**1.20/ind**
Fluconazole	0.60	0.50	0.40	0.30	0.50	0.20	0.15	0.20	0.20
AVG	0.60	0.50	0.40	0.30	1.00	0.20	0.60	1.60	0.80
**FICi**	**1.20/ind**	**1.00/ind**	**0.80/ind**	**0.60/ind**	**1.50/ind**	**0.40/syn**	**0.75/ind**	**1.80/ind**	**1.00/ind**
Ketoconazole	0.20	0.40	0.30	0.25	0.15	0.20	0.50	0.30	0.40
AVG	0.40	0.40	0.60	0.50	0.60	0.40	1.00	1.20	0.80
**FICi**	**0.60/ind**	**0.80/ind**	**0.90/ind**	**0.75/ind**	**0.75/ind**	**0.60/ind**	**1.50/ind**	**1.50/ind**	**1.20/ind**
Terbinafine	0.10	0.20	0.60	0.40	0.60	1.20	1.00	1.60	0.30
AVG	0.40	0.40	0.60	0.20	0.60	0.30	1.00	1.60	0.60
**FICi**	**0.50/syn**	**0.60/ind**	**1.20/ind**	**0.60/ind**	**1.20/ind**	**1.50/ind**	**2.00/ind**	**3.20/ind**	**0.90/ind**

*syn* Synergetic, *ind* Indifferent

^a^Bold indicates Fractional Inhibitory Concentration index (FICi) / their interpretation

## Discussion

Researchers across the board have responded to continuous increase of multiple antibiotic resistance by pathogenic fungal strains and thus, revealed that novel potential strategy to promote antifungal therapeutic is urgently needed to be explored [26]. In this study, the combinatory potential of AFCs with TEO or AVG was assessed. Terbinafine was efficient AFC against pathogenic fungi in vitro. Terbinafine is known as a broad spectrum antifungal agent, active against wide range of dermatophytes, moulds, yeasts and dimorphic fungi [27]. However, studies by Karri et al. [28] reported less activity of terbinafine against *Candida albicans*. Terbinafine was considered to have potency against dermatophytes but now, there is a rise to terbinafine resistance by pathogenic fungi [29].

The efficacy of AFCs (azole creams) such as clotrimazole, fluconazole, and ketoconazole against selected pathogenic fungi was observed, while fungi such as *Candida albicans, Trichophyton rubrum, T. mentagrophytes* required higher concentration of AFCs before being inhibited. Shivamurthy et al. [30] reported that sertaconazole showed better anti-dermatophytic in clinical parameters than topical clotrimazole within a span of 3 weeks in the treatment of *Tinea corporis*. Sabatelli et al. [31] tested triazoles against wide number of clinically important pathogenic fungi (19,000 yeast and mould) and found out that, species of *Candida* and *Aspergillus* exhibited resistance to fluconazole, voriconazole, itraconazole and amphotericin B except posaconazole that was more efficient. Azole or triazole are commonly used antifungal agents that suppress fungi growth by inhibiting a key enzyme; lanosterol 14alpha demethylase, which occurs through the binding of the free nitrogen atom of the azole ring to the iron atom of the heme-group of the enzyme [32].

In this study, in vitro antifungal effectiveness of over-the-counter AFCs and their synergism with TEO or AVG against pathogenic fungi of clinical sources was attributed to phytochemicals as well as bioactive ingredients in TEO and AVG. Phytochemicals are biologically active, naturally occurring chemical compounds in plants that promote human health and prevent diseases [33]. The presence of these biologically active phytochemicals (phenol, flavonoids, saponins, alkaloids, steroids, terpenoids, cardiac glycosides, anthraquinones and tannins) in studied extracts make them useful for some medicinal purposes such as antimicrobial against pathogenic microorganisms. Sawant and Godghate [34] reported that turmeric was one of the best source to obtain a variety of drugs due to its rich phytochemical constituents.

Bawankar et al. [35] reported the presence of hexadecanoic acid, 1-(phenylthioxomethyl) piperidine, 6-hydroxyhexane-3-1, octadecanoic acid, tricosane, 1-octadecanol, stigmasterol, docosane in the ethanolic extract of *A. vera*. Hydroxybenzhydrazide was found in AVG. It is a hydroxylated phenolic compound with strong and moderate antimicrobial activity [36]. Hydroxylated phenolic compounds like pyrocatechol are known to be toxic to microorganisms [37]. The toxicity of phenolic compounds to microorganisms are believed to be related to the number of hydroxyl groups, which inhibit microbial growth by cell membrane disruption and protein denaturation [38]. Alpha-phellandrene, α-pinene, myrcene, linalol, geraniol, ar-turmerone, turmerone, α-curcumene, zingiberene, turmerones, curcuminiods, geraniol acetate beta-sesquiphellandrene, (2E,6E)-farnesol, camphene, tricyclen cineole were major bioactive compounds as aromatic compounds in EOs with dynamic antimicrobial features [39, 40]. Alpha-phellandrene is a terpene-derivative metabolite, which is mostly found in volatile oils and plays a role of an antimicrobial agent [41]. The presence of α-phellandrene in this study correlates with the findings of Mukesi et al. [42] who examined the bioactivity of commercial antimicrobials, EO and ethanolic extracts of *Olea europaea*. Another major component found in TEO is 2-carene. It is a bicyclic monoterpene that occurs in several EOs with a sweet and pungent odour. Carene and its derivatives are of modest relevance in the perfume industry [43], hence, its presence in TEO could contributed to spicy aromatic scent. Farnesene, a volatile compound, which was identified in TEO is known to be responsible for the characteristic taste and flavour of turmeric related to peppermint [44].

TEO exhibited pronounce inhibition against *Candida albicans, Penicillium notatum,* species of *Aspergillus* and *Trichophyton*. Ferreira et al. [45] attributed the inhibition of *A. flavus* growth at 0.10%, reduction of their viable spores at 0.10% and complete inhibition at 0.50% to ar-turmerone *α*-turmerone and *β*-turmerone, being a major component of EO of *C. longa*. EOs components act as antifungal agents (fungistatic and fungicidal) against fungi by deactivating or disrupting the structure and function of membranes or organelles of fungal cell and/or inhibiting the nuclear material or protein synthesis inactivation, inhibition of intracellular and extracellular enzymes [38].

The antagonistic activity of *A. vera* against bacteria, fungi and viruses has been expounded by some studies [46–48]. The antifungal potential of AVG against tested pathogenic fungi corresponds to the findings of Nidiry et al. [49] who reported antifungal property of bioactive constituents; aloin and aloe-emodin in *A. vera* against *Colletotrichum gloeosporides* and *Cladosporium cucumerinum*. The inhibitory efficiency of AVG corroborates to the findings of Khwakhali and Shrivatava [50] who reported the effectiveness of *A. vera* against pathogenic *Aspergillus* spp., while Al-Snafi [51] obtained effective treatment (70% growth inhibition) of guinea pig infected with *T. mentagrophytes*. AVG possessed broad antifungal activities against the tested fungi. Findings of Bawankar et al. [35], Saks and Barkai-Golan [52], and Yebpella et al. [53] have reported antifungal activity of AVG against the growth of *Penicillium* spp.*, Botrytis cineria, Alternaria alternate, Aspergillus* spp*.* and *Candida albicans* at varying concentrations. The antimicrobial potential of *A. vera* could be attributed to anthraquinone and pyrocatechol in the gel of leaves, which are toxic to microorganisms by blocking their ribosomal A site [54]. *A. vera* is one of the most important traditional folk and alternative medicine often used for the treatment of infectious diseases with no side effects [55].

In this study, it was observed that the combination of plant extracts; TEO or AVG with AFC was effective against all the tested fungi. Jankasem et al. [56] revealed that turmeric oil displayed better anti-dermatophytic activity with the MICs of 1.56–6.25 *μ*g/mL when compared to 3.90–7.81 *μ*g/mL of ketoconazole. Shin and Lim [57] revealed that antifungal potential of ketoconazole was significantly improved against *Trichophyton schoenleinii, T. erinacei and T. soudanense* when combined with EO of *Pelargonium graveolens*. The FICi obtained for oils of thyme, cinnamon, clove and eucalyptus combined with amphotericin B against *C. albicans* and *A. niger* suggested that synergistic of antifungal drugs with herbs (oil or and extracts) yielded efficacious dose for the treatment of fungal infections and thus, minimizing its side effects [58]. Most EO of *Styrax tonkinensis, Lavandula angustifolia, Melaleuca alternifolia, Rosmarinus officinalis,* and *Pelargonium graveolens* and its fractional components; geraniol and citronellol exhibited additive effect when combined with amphotericin B and with ketoconazole against *Aspergillus* spp., which resulted to FICi ranged from 0.52 to 1.00 [53]. In the findings of Scalas et al. [40], EOs of *Origanum vulgare* (oregano), *Pinus sylvestris* (pine), and *Thymus vulgaris* (thyme red) and their components (α-pinene, carvacrol, thymol) exhibited good antifungal activity against *Cryptococcus neoformans* strains compared to fluconazole, itraconazole, and voriconazole, and thus, revealed the synergistic and additive for EO and azole (itraconazole) combination. Potential synergistic combination between two or more antimicrobial agents help in reducing resistant mutants, exhibit more antimicrobial action, toxicity against pathogens and thus, serve as effective alternative traditional medicine for the treatment of various fungal infections [59–61]. The use of EOs aromatic compounds and plant extracts in formulation of topical AFCs need to be embraced to achieve optimal antifungal activity with no side effects. The availability of natural products (EOs or plant extracts) and development of combined antimicrobial agents are often an optional therapy for dermatological infections [62].

## Conclusion

AFCs, TEO and AVG inhibited the growth of all tested pathogenic fungi with varying degrees of zones of inhibition. Combinatory action of AFCs with TEO or AVG did not slow down their bioactivity against tested fungi. This indicated that bioactive compounds in plant extracts can complement the activity of AFCs to improve their clinical efficacy. The antifungal properties of TEO or AVG combined with different AFCs established their importance in phytomedicine and cosmeceutical. The combination of AFCs with plant extracts will serve as alternative medicine in treating or combating many infectious fungal diseases such as dermatophytosis, which had been a widespread disease. The bioactive ingredients in plant extracts could argument the formulation of body and hair creams (cosmetics) to treat resistant pathogenic fungi within short time with no side effects.

## Data Availability

The data used or analysed during the current study are available.
